# Six Years in the Life of a Mother Bear - The Longest Continuous Heart Rate Recordings from a Free-Ranging Mammal

**DOI:** 10.1038/srep40732

**Published:** 2017-01-17

**Authors:** Timothy G. Laske, Paul A. Iaizzo, David L. Garshelis

**Affiliations:** 1 University of Minnesota, Department of Surgery, Minneapolis, 55455, USA; 2 Minnesota Department of Natural Resources, Grand Rapids, 55744, USA

## Abstract

Physiological monitoring of free-ranging wild animals is providing new insights into their adaptations to a changing environment. American black bears (*Ursus americanus*) are highly adaptable mammals, spending up to half the year hibernating, and the remainder of the year attempting to gain weight on a landscape with foods that vary seasonally and year to year. We recorded heart rate (HR) and corresponding activity of an adult female black bear over the course of six years, using an implanted monitor. Despite yearly differences in food, and an every-other year reproductive cycle, this bear exhibited remarkable consistency in HR and activity. HR increased for 12 weeks in spring, from minimal hibernation levels (mean 20–25 beats/minute [bpm]; min 10 bpm) to summer active levels (July daytime: mean 95 bpm). Timing was delayed following one cold winter. In August the bear switched from primarily diurnal to nocturnal, coincident with the availability of baits set by legal hunters. Activity in autumn was higher when the bear was with cubs. Birthing of cubs in January was identified by a transient increase in HR and activity. Long-term physiological and behavioral monitoring is valuable for understanding adaptations of free-ranging animals to climate change, food availability, and human-related stressors.

American black bears (*Ursus americanus*) are one of the most physiologically remarkable mammals^1^,^2^. In most of their ranges, their food supply disappears over the winter forcing them to spend 4–6 months in a state of dormancy, with minimal physical activities, in a state of mild hypothermia (~30–36 °C), without eating or drinking and typically without urinating or defecating^3^. Hibernating females give birth during mid-winter and also den with these cubs in the subsequent year^4^. Some bears spend the winter in either partially-exposed dens or open nests with associated higher risks for predation and/or external disturbance^5^,^6^. Importantly, hibernating bears can defend themselves and their offspring because of their unique maintenance of muscle strength and their capability of rapidly rousing from hibernation when a threat is present^7^,^8^,^9^. Black bears commonly elicit defensive posturing and high respiratory rates upon disturbance, with their heart transitioning from the quiescent state of hibernation to sustained elevations, so to support bursts of activity^10^.

During the active part of the year, black bears attempt to regain the weight lost during hibernation and put on weight in preparation for the next winter’s fast^4^,^11^,^12^. Although black bears are omnivores, most of their diet is vegetation (greens, fruits, and nuts), which is variable seasonally and year to year^13^,^14^. Moreover, they are highly opportunistic and adaptable, taking advantage of landscapes with both natural and human-related sources of food^15^,^16^. Black bear populations in the United States, Canada and Mexico have been expanding geographically, resulting in more frequent interactions with humans^17^,^18^.

Although important physiological data have been obtained in both captive and wild bears, long-term monitoring has been limited by the size, memory storage, system functionality, and/or battery longevity of recording devices^19^,^20^,^21^,^22^,^23^,^24^,^25^,^26^,^27^. Through the use of serial devices, here we report the recording of 6 years of heart rate and activity monitoring from an adult female American black bear living in northern Minnesota. This is a region where weather and food supplies are especially variable seasonally and year to year. Our aim was to examine seasonal patterns, yearly differences, and specific events in the life of this individual bear which could serve as a model to demonstrate the value of such physiological monitoring in free-ranging animals.

## Results

**Figure 1:**
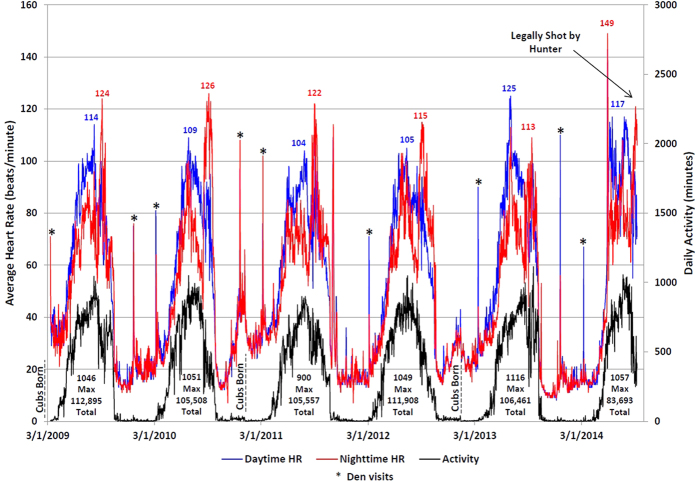
Average daytime and nighttime HR and total daily activity in each of six years for an adult female black bear in northern Minnesota. Peak daily values are shown each year. Cubs were born in January of 2009, 2011, and 2013 and remained with the mother for 17 months. Asterisks denote den visits by the research team: the heart rate was elevated due to the effects of the anesthetic agent which eliminates autonomic tone and the respiratory sinus arrhythmia.

**Figure 2:**
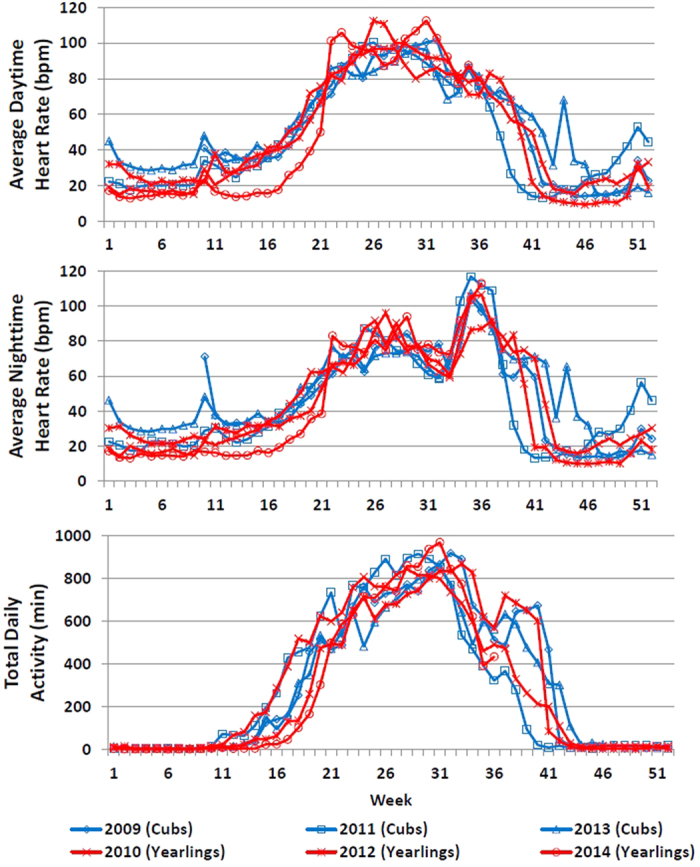
Weekly averages for daytime HR, nighttime HR, and total daily activity for a female black bear, demonstrating year to year consistency. The bear had year-old cubs (yearlings) in the spring of alternate years, and was solitary in summer and autumn of these years. Increasing HR and activity occurred throughout the spring, but was delayed in 2014 following a colder winter. (The period of January-March in 2014 was colder than the same period in all other periods; p < 0.0004 for all comparisons).

Recordings of heart rate (HR) and levels of activity were obtained from March 10, 2009 to September 1, 2014 (2002 days) in an adult female American black bear (#2213; the “Spider Lake bear”). This time period included parturition and the rearing of 3 litters of cubs (born in January of 2009, 2011, and 2013). Cubs remained with their mother through one winter and the following spring, at which time the yearlings separated and she again mated; thus, she was solitary only during the summers and autumns of 2010, 2012, and 2014. Figure 1 plots the daytime HR, nighttime HR, and daily amount of activity for each of the 6 years; Fig. 2 displays the same dataset as weekly averages overlaid for the 6 years to demonstrate the remarkable consistency in patterns and levels of HR and activity. Table 1 summarizes a number of parameters relating to HR, behaviors, and variations in weather. Table 2 provides the monthly summaries for daytime HR, nighttime HR, and daily activity for the entire time period, noting years with cubs and yearlings.

Over the 6 consecutive years, HR steadily increased over roughly a 12 week period each spring from hibernation levels (20–25 beats/minute (bpm)) to summer active levels (July daytime HR averaged 95 bpm and nighttime HR averaged 78 bpm). Maximum average daytime HR ranged from 104–125 bpm and maximum average nighttime HR ranged from 113–149. Daily activity was as low as 0 minutes/day during hibernation, averaging <12 minutes/day, with maximum daily activity ranging from 900–1116 minutes (15.0–18.6 hours) per day. Total annual activity was remarkably consistent, ranging from 105,508–112,895 minutes of movement/year (1,758–1,882 hours/year; averaging 13.5 hours/day in July).

Emergence from hibernation and departure from the den site, as evidenced by consistent activity and sustained HRs of greater than 40 bpm, occurred from 25-Apr to 04-May in 5 of the years. This occurred later in 2014 (17-May) presumably due to the colder weather that year (colder than all other periods of Jan-Mar; p < 0.0004 for all comparisons of daily averages; 2014 Jan-Mar mean = −14.2 C, min = −30 C, max = 4.4 C). Presumed denning in the fall, as evidenced by sustained HRs of less than 40 bpm, occurred during 23-Sep to 23-Oct. Each year, the bear switched from primarily diurnal activities to primarily nocturnal: this switch occurred between 22-Jul and 24-Aug, and was on nearly the same day in 4 of 6 years (13-Aug, 13-Aug, 16-Aug, and 18-Aug; Table 1).

We identified dates of parturition in 2011 and 2013 (04-Jan and 11-Jan, respectively). (See Supplementary Video V1.) Parturition was marked by increased HR during the second week of January (daytime average HRs = 32.5 v. 16.6 bpm in alternate years, p = 0.015; average nighttime HRs = 32.4 v. 16.1 bpm in alternate years, p = 0.008). Other statistically significant differences included higher average daytime HRs during March with newborn cubs than with yearlings (33.7 v. 21.3 bpm, p = 0.045), higher average daytime HRs in June when solitary (77.4 v. 70.8 bpm, p = 0.010), and more active in July when solitary (856 v. 768 minutes/day, p = 0.019), but more active in September with cubs (602 v. 349 minutes/day, p = 0.014).

**Figure 3:**
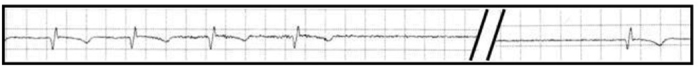
Respiratory sinus arrhythmia and long sinus pause recorded from female black bear #2213. An electrocardiogram (ECG) is shown for 18-Dec-2009 at 12:21. A 14.4 second pause occurred following acceleration in heart rate with inspiration.

**Figure 4:**
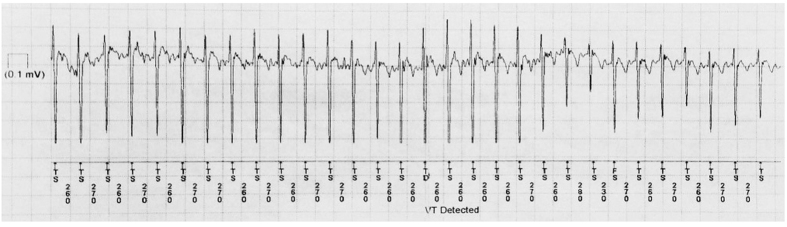
An electrocardiogram from female black bear #2213 on 07-Sep-2011 recording a ventricular tachycardia with an average heart rate of 227 beats/minute and a maximum heart rate of 231 beats/minute. This episode occurred during the legal hunting season in 2011, but whether it was related to a human interaction is unknown. The interval between consecutive heart beats is shown at the bottom of the strip recording (260 milliseconds = 231 bpm). Tachycardia detection occurred when 16 consecutive heart beats exceed the programmed detection setting of 176 bpm, at which time the device stored the associated ECG in memory.

**Figure 5:**
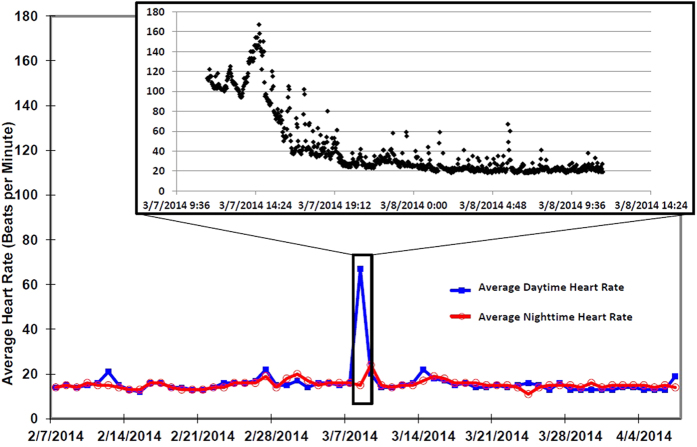
Heart rate recordings of a female black bear in northern Minnesota during a visit to her den by the research team. A two month interval of daily average daytime and nighttime HR is plotted, with a 12 hour window inset (07-Mar-2014) showing the average HR every two minutes (plot starts at the time of the team’s departure at 11:30 AM). The HR returned to pre-visit levels typical of winter hibernation within approximately 6–8 hours.

Extreme respiratory sinus arrhythmias were elicited during winter months, with HRs as low as 10 bpm, sinus pauses of up to 14.4 seconds, and respiratory rates as low as 1.5 breaths/min (Fig. 3). By contrast, during summer active periods, HRs as high as 231 bpm were recorded (Fig. 4). During our visits to the bear’s denning sites while she was hibernating, the devices recorded transient spikes in HRs (Fig. 1) likely due to both the effects of the anesthetic agents and the presence of our research team (pre-anesthesia). The HR increases associated with a typical visit is shown in Fig. 5, with rates returning to those of undisturbed hibernation within 6–8 hours.

**Figure 6:**
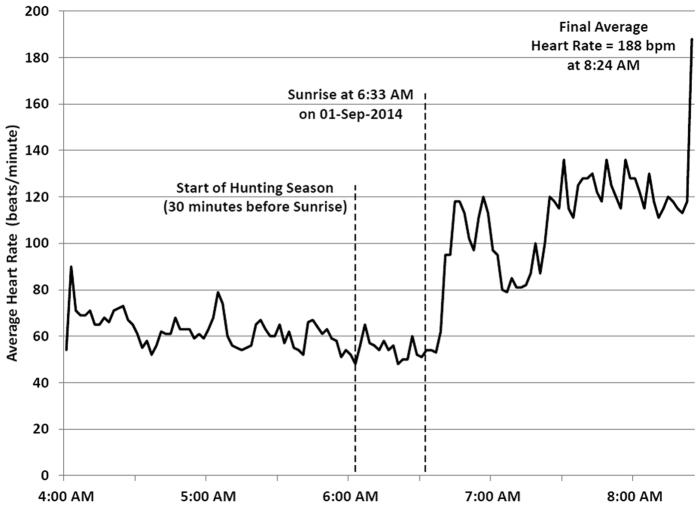
Heart rate recording of female black bear legally shot on the opening morning of hunting season on September 01, 2014. Her heart rate prior to sunrise averaged approximately 60 bpm, then elevated to 120 bpm, with a final average rate of 188 bpm at 8:24 AM.

Device monitoring ended when this bear was legally shot and killed by a hunter in September, 2014. Her HR before sunrise averaged 60 bpm, then increased to about 120 bpm for an hour; her final HR averaged 188 bpm (Fig. 6).

## Discussion

Here we present the longest continuous recording of HR and associated activity in a free-ranging mammal. These data provide novel insights into the extreme physiological and behavioral changes that occur seasonally in this large mammalian species, with this female bear serving as her own control. Similar overall patterns in annual HRs were recorded over the 6 years, including: (1) steadily increasing HR and activity levels post-hibernation for a period of approximately 12 weeks in spring; (2) an abrupt and consistent switch from primarily diurnal to nocturnal behavior in late summer; (3) sharply decreased activity in fall; (4) extraordinary low activity and HRs for 7 months (Sep-Oct through April-May) associated with denning; and (5) significant changes in HR and activity associated with parturition. These yearly trends in HR and activity seemed to correspond to caloric availability in her environment^11^,^12^,^13^. We anticipated seeing differences in activity and energy expenditure related to year to year differences in food production, but these were not clearly evident. The summer of 2012 was a poor year for natural fruit production – in fact the lowest production in 17 years – whereas 2013 and 2014 were above-average [Minnesota Department of Natural Resources, Unpublished Data]. However, this bear seemed to adapt to these radical fluctuations in food with no apparent changes in her summer activity or HR. We were able to detect one outlier year (2014) for den emergence, apparently related to weather conditions. This finding points to the value of HR and activity monitoring for perceiving effects of short-term and long-term effects of climate change. With warming trends, we might expect to see later denning, earlier den emergence and possibly earlier parturition to enable cubs to leave the den earlier, and more pulses of activity in mid-winter due to unusually warm weather and melting snow^28^,^29^,^30^.

The increased HRs and activity levels in summers when the bear was solitary might be indicative of interactions with males associated with mating. Conversely, the higher levels of activity when she was with cubs in September might be indicative of increased foraging necessary to satisfy the family group prior to hibernation. We are unsure of the reason for the abrupt switch to increased nocturnal activity each year. The date of this switching, though, corresponds with setting of baits by hunters in mid-August (2–3 weeks before the start of actual hunting on 1-Sep), suggesting that this bear attempted to avoid people while feeding at human-related food sources, as has been commonly observed elsewhere^31^,^32^,^33^: indeed, hunters captured photos of her with camera-traps at their baits, and she was eventually shot at a bait. It is worth noting that the episode presented in Fig. 4 with a peak HR of 231 bpm occurred during the hunting season of 2011.

Matching HR and activity with habitat and movements (e.g., using GPS radio collars) would enable more insights into reasons for changes in the animal’s behavior^27^,^34^. Additionally, more detailed investigations of the unique physiology of hibernating bears may also provide novel therapeutic approaches in human medicine^35^,^36^,^37^.

## Methods

A wild radiocollared female bear in northern Minnesota (Identification #2213, born in year 2000) was located in her winter den, anesthetized (Telazol^®^, veterinary formulation of tiletamine and zolazepam; averaging 4.5 mg/kg) and temporarily extricated either once or twice per year over a period of six years. Ethylene Oxide sterilized Insertable Cardiac Monitors (ICMs; Reveal^®^ XT, Model 9529; Medtronic Inc., Minneapolis, MN; 9 cc; 8 mm × 19 mm × 62 mm; 15 grams) were surgically implanted at the den site in a peristernal location using aseptic techniques^10^. Following device implantation and/or retrieval of data via wireless telemetry, she was returned to her original location in her winter den sites with either her cubs or yearlings.

The ICMs utilized for this research were indicated for human clinical use. The electronics of these devices were housed in hermetically sealed titanium cans. Electrocardiograms (ECGs) were recorded using the differential voltage measured between a titanium electrode housed in a polyurethane and silicone header and an exposed region on the parylene coated titanium can. A built-in single axis accelerometer detected movement and was used to document the number of minutes of activity in a given period. Device programming and data retrieval is non-invasive via transcutaneous telemetry associated with a programming system (CareLink Model 2090 Programmer with software Model SW007, Medtronic Inc., Minneapolis, MN). In addition to storing HR and activity data over the three year life of each device, these ICMs stored up to 27 min of ECG recordings from automatically detected arrhythmias. The devices report average daytime heart rate (HR) (08:00–20:00; referencing a 24 hour clock) and average nighttime HR (0:00–04:00) and total daily amount of activity in minutes. The devices used in the final year had a custom software version that also recorded average HR every 2 minutes and total activity in each 15 minute interval. Devices were programmed in the field to automatically detect and store episodes when the HR exceeded 176 beats/minute (bpm) or when there was no detected heartbeat for a time ≥4.5 seconds (asystoles). The same programmer was used to non-invasively download data during subsequent visits.

Studies were approved and governed by the University of Minnesota’s Institutional Animal Care and Use Committee under Protocol #1411–32045 A and were conducted in accordance with all relevant guidelines and regulations. All statistical analyses were performed using a two-tailed t-test assuming unequal variances. P-values less than or equal to 0.05 were considered significant.

## Additional Information

**How to cite this article:** Laske, T. G. *et al*. Six Years in the Life of a Mother Bear - The Longest Continuous Heart Rate Recordings from a Free-Ranging Mammal. *Sci. Rep.* **7**, 40732; doi: 10.1038/srep40732 (2017).

**Publisher's note:** Springer Nature remains neutral with regard to jurisdictional claims in published maps and institutional affiliations.

## Supplementary Material

Supplementary Information

Supplementary Video

## Figures and Tables

**Table 1:** Tabulation of physiological and behavioral parameters and weather recorded during six years in the life of female black bear #2213 in northern Minnesota.

	Maximum Daily Average Daytime HR (bpm)	Maximum Average Daily Nighttime HR (bpm)	Maximum Daily Activity (minutes)	Total Annual Activity (minutes)	Estimated Date of Parturition^b^	Initiation of Activity in the Spring^c^	Cessation of Activity in the Fall^d^	Diurnal to Noctural Switching^e^	Temperature During Late Hibernation^f^ (Mean/Min/Max in °C)
2009 (cubs born in January)	114 (07-Aug-2009)	124 (03-Sep-2009)	1046 (06-Aug-2009)	112,895	Pre-initiation of recording	04-May	11-Oct	24-Aug	−9.8
									−27.2
									7.8
2010	109 (26-Jun-2010)	126 (03-Sep-2010)	1051 (26-Jun-2010)	105,508	N/A	25-Apr	23-Sep	16-Aug	−6.2
									−25.0
									14.4
2011 (cubs born in January)	104 (27-Jul-2011)	122 (30-Aug-2011)	900 (18-Jul and 30-Jul-2011)	105,557	04-Jan-2011	26-Apr	23-Oct	13-Aug	−9.6
									−27.8
									5.6
2012	105 (11-Jul-2012)	115 (01-Sep-2012)	1049 (14-Jul-2012)	111,908	N/A	26-Apr	18-Oct	13-Aug	−3.1
									−22.8
									18.9
2013 (cubs born in January)	125 (01-Jul-2013)	113 (02-Jul-2013)	1116 (17-Sep-2013)	106,461	11-Jan-2013	02-May	5-Oct	22-Jul	−10.1
									−26.7
									5.6
2014	117 (14-Jun and 25-Jul-2014)	149 (29-May-2014)	1057 (21-Jul-2014)	83,693^a^	N/A	17-May	N/A^a^	18-Aug	−14.2
									−30.0
									4.4

Daytime HR = 08:00–20:00, Nighttime HR = 00:00–04:00, HR = heart rate, bpm = beats per minute, N/A = not applicable.

^a^Partial year (shot and killed on 01-Sep-2014).

^b^Based-upon increase in HR and activity.

^c^First day that the average daytime HR was sustained at over 40 bpm.

^d^First day that the average daytime HR was sustained at less than 40 bpm.

^e^First day that average nighttime HR exceeded the daytime HR for 2 days or more.

^f^Ambient temperatures from 01-Jan to 31-Mar in Grand Rapids, MN (www.wundergound.com).

**Table 2:** Tabulation of physiological and behavioral parameters recorded during six years in the life of female black bear #2213 in northern Minnesota.

	All Years Combined (2009–2014)						Years with Cubs (2009, 2011, 2013)						Years with Yearlings (2010, 2012, 2014)					
	Average Daytime Heart Rate (bpm)		Average Nighttime Heart Rate (bpm)		Total Daily Activity (minutes)		Average Daytime Heart Rate (bpm)		Average Nighttime Heart Rate (bpm)		Total Daily Activity (minutes)		Average Daytime Heart Rate (bpm)		Average Nighttime Heart Rate (bpm)		Total Daily Activity (minutes)	
Month	Avg.	Std. Dev.	Avg.	Std. Dev.	Avg.	Std. Dev.	Avg.	Std. Dev.	Avg.	Std. Dev.	Avg.	Std. Dev.	Avg.	Std. Dev.	Avg.	Std. Dev.	Avg.	Std. Dev.
Jan	22.55	7.88	22.55	7.74	4.25	2.18	30.52	4.38	30.39	4.70	6.44	1.03	17.25	2.97	17.32	2.55	2.80	0.99
Feb	20.90	5.92	21.02	6.10	2.81	1.08	26.27	5.68	26.36	5.61	3.59	1.39	17.32	2.42	17.46	3.34	2.30	0.62
Mar	28.86	7.85	27.52	8.28	22.29	18.51	34.35	5.33	33.72	5.38	12.76	1.34	23.37	5.92	21.31	5.18	31.82	24.13
Apr	33.69	8.75	30.27	6.71	144.48	97.78	37.58	2.68	33.53	2.10	105.23	27.69	29.80	11.79	27.01	8.73	183.72	136.07
May	60.47	6.78	50.63	6.40	457.09	101.16	61.73	4.57	50.75	6.13	423.01	36.34	59.20	9.45	50.51	8.04	491.16	144.15
Jun	91.07	5.70	74.11	3.88	701.34	72.88	87.92	5.91	70.84	1.84	648.50	52.50	94.22	4.06	77.37	1.56	754.18	46.32
Jul	95.45	4.04	77.90	4.18	811.60	53.18	96.67	4.19	77.51	4.65	767.57	18.21	94.24	4.34	78.29	4.65	855.63	30.38
Aug	84.90	5.33	78.06	4.15	696.18	83.61	84.35	5.43	76.37	3.29	745.35	77.93	85.44	6.37	79.75	4.87	647.00	64.41
Sep	70.50	9.24	87.47	13.48	475.51	151.02	73.40	1.48	81.14	3.10	601.52	56.11	67.60	13.64	93.80	18.01	349.49	78.94
Oct	30.28	13.25	35.95	17.39	152.50	98.84	33.83	14.29	39.59	17.29	211.99	59.75	24.97	14.14	30.48	22.63	63.27	73.47
Nov	20.01	8.33	19.73	8.76	11.59	7.05	19.06	11.57	19.09	12.25	9.94	9.37	21.45	1.72	20.68	1.86	14.07	1.70
Dec	25.50	10.42	24.54	10.89	10.37	5.33	19.23	2.86	18.04	2.59	6.60	0.99	34.90	11.09	34.27	12.02	16.03	2.19

Daytime HR = 08:00–20:00, Nighttime HR = 00:00–04:00, bpm = beats per minute, Avg. = average, Std. Dev. = standard deviation.
