# Supplementary material for: Six Years in the Life of a Mother Bear - The Longest Continuous Heart Rate Recordings from a Free-Ranging Mammal
Source: Sci Rep. 2017 Jan 17;7:40732. doi: 10.1038/srep40732 (PMC5240622; doi:10.1038/srep40732)
Supplement: Supplementary Information [file srep40732-s1.pdf]

Six Years in the Life of a Mother Bear - The Longest Continuous Heart Rate Recordings from a Free-Ranging Mammal

Timothy G Laske, Paul A Iaizzo, and David L Garshelis

Supplementary Video V1: Female black bear #2213 and her cubs hibernating in an open nest in northern Minnesota (March 2009).
